# 

**Published:** 1892-04

**Authors:** 


SUBSCRIBE FOR THE

### International Dental Journal

### The Organ of the Profession,

PUBLISHED BY THE

### INTERNATIONAL DENTAL PUBLICATION COMPANY:

**LOUIS JACK,** *President*, Philadelphia.

**BENJAMIN LORD,** *Vice-President*, New York.

**C. N. PEIRCE,** *Treasurer*, Philadelphia.

**GEO. S. ALLAN,** *Secretary*, 51 W. 37th St., New York

---

*PUBLICATION OFFICE:*

716 Filbert Street, Philadelphia, Pa.

---

### FOREIGN CORRESPONDENTS:

Dr. ARKÖVY, Budapesth, Hungary.
W. GEO. BEERS, D.D.S., Ed. Dominion Dental Journal, Montreal.
L. C. BRYAN, D.D.S., Basel.
ALFRED BURNE, D.D.S., Sydney, New South Wales.
F. BUSCH, M.D., Berlin.
GEORGE CUNNINGHAM, Cambridge, Eng.
I. B. DAVENPORT, D.D.S., Paris.
PAUL DUBOIS, Ed. L'Odontologie, Paris.
WILLIAM DUNN, Jr., L.D.S., Florence.
A. H. EDWARDS, D.D.S., Madrid.
W. ST. GEORGE ELLIOTT, M.D., D.D.S., Lon.
OSWALD FERGUS, D.D.S., Glasgow, Scotland.
ALPHONSE FEUCHEL, Zahnarzt, St. Petersburg.
C. R. FLETCHER, D.D.S., Sao Paulo, Brazil.
WALTER HARRISON, L.D.S., D.M.D., Brighton, Eng.
ROBERT S. IVY, D.D.S., Shanghai, China.
N. S. JENKINS, D.D.S., Dresden.
F. KLAPPROTH, Zahnarzt, St. Petersburg.
L. KOLBE, " " "
Dr. GEO. P. E. KUHN, Paris.
W. BOWMAN MACLEOD, L.D.S., Edinburgh.
W. D. MILLER, Ph. D., M.D., D.D.S., Berlin.
WILLIAM SACHS, D.D.S., Breslau.
J. G. VAN MARTER, B.A., D.D.S., Rome.
J. M. WHITNEY, M.D., D.D.S., Honnolula.
LUDWIG WARNEKROS, Zahnarzt, Berlin.
LUDW. ADOLPH WEIL, M.D., Munich.
J. COWAN WOODBURN, M.D., Glasgow.

---

### STOCKHOLDERS:

Frank Abbott,
Geo. S. Allan,
W. W. Allport,
R. R. Andrews,
H. A. Baker,
A. E. Baldwin,
W. C. Barrett,
A. P. Beale,
S. T. Beale, Jr.,
C. S. Beck,
G. V. Black,
C. F. W. Bödecker,
E. A. Bogue,
W. G. A. Bonwill,
C. A. Brackett,
Thomas Bradley,
Edward C. Briggs,
Albert H. Brockway
A. E. Brown,
Wm. Carr,
Geo. H. Chance,
G. F. Cheney,
Dwight M. Clapp,
John D. Codman,
Chas. D. Cook,
J. N. Crouse,
Edw. T. Darby,
H. S. Draper,
Wm. H. Dwinelle,
A. R. Eaton,
Chas. J. Essig,
J. N. Farrar,
T. Fillebrown,
W. B. Finney,
T. A. Fletcher,
M. W. Foster,
Chas. E. Francis,
E. C. Fundenberg,
W. F. Fundenberg,
W. H. Fundenberg,
E. S. Gaylord,
J. P. Geran,
A. H. Gilson,
S. H. Guilford,
John I. Hart,
O. E. Hill,
J. F. P. Hodson,
J. Morgan Howe,
Robert Huey,
A. O. Hunt,
Louis Jack,
V. H. Jackson
C. R. Jefferis,
C. A. Kingsbury,
C. R. E. Koch,
W. T. La Roche,
W. K. Leech,
F. A. Levy,
Wilbur F. Litch,
J. Bond Littig,
Benjamin Lord,
Geo. W. Lovejoy (Canada),
John S. Marshall,
H. J. McKellops,
Charles McManus,
James McManus,
Dan'l Neal McQuillan,
Horatio C. Meriam,
W. D. Miller (Berlin),
W. E. Page,
S. B. Palmer,
C. B. Parker,
D. D. Peabody,
C. N. Peirce,
S. G. Perry,
W. A. Phreaner,
Chas. E. Pike,
W. E. Pinkham,
W. H. Potter,
Chas. P. Pruyn,
H. C. Register,
F. A. Roy,
Z. T. Sailer,
L. D. Shepard,
Geo. R. Shidle,
Eugene H. Smith,
B. Holly Smith,
H. A. Smith,
S. G. Stevens,
W. X. Sudduth,
Eugene S. Talbott,
Ambler Tees,
J. D. Thomas,
James Truman,
Wm. H. Trueman,
W. W. Walker,
I. F. Wardwell,
G. W. Warren,
T. S. Waters,
Geo. W. Weld,
Julius G. W. Werner,
Jacob L. Williams,
R. B. Winder,
C. A. Woodward,
J. A. Woodward,
W. A. Woodward,
W. J. Younger.

---

***Subscription Price, $2.50 per Year.***

### IMPORTANT NOTICE.

For the convenience of subscribers to the INTERNATIONAL DENTAL JOURNAL, who often find it difficult to remit small amounts through the mail, we have established agencies in different localities (in most cases with dealers in Dental Goods, with whom dentists have frequent business transactions), WHERE EITHER NEW OR RENEWAL OF SUBSCRIPTIONS MAY BE PAID.

### International Dental Publication Co.,

PUBLISHERS.

### LIST OF AGENTS.

| NAME. | ADDRESS. | BUSINESS. |
|---|---|---|
| C. Ash & Sons | 30 East 14th St., New York | Dental Goods. |
| E. Steiger & Co. | 32 Park Place, New York. | |
| Fred. W. Smith | Binghamton, N. Y. | Dental Depot. |
| J. H. A. Folkers & Bro. | 815 Market Street, San Francisco, Cal. | |
| Osman & Cook | Newark, N. J., Box 265 | Newark Dental Depot. |
| B. L. Knapp & Co | 161 Tremont St., Boston, Mass. | Dentists' Materials. |
| Stuart & Adams | New Orleans, La. | Dental Depot. |
| Dr. F. W. Seabury | 294 Westminster Street, Providence, R. I. | Dentist. |
| E. L. Washburn & Co. | New Haven, Conn. | Dental Depot. |
| Dr. A. W. Clark | Jefferson, N. Y. | Dental Supplies. |
| Keller Dental Co | Fort Wayne, Ind. | Dental Manufacturers. |
| Ransom & Randolph | Toledo, Ohio | Dental Depot. |
| John Rowan | St. Louis, Mo. | Dental Depot. |
| Lee S. Smith | 52 6th St., Pittsburgh, Pa. | Dental Depot. |
| L. J. Frazel | 438 W. Walnut Street, Louisville, Ky. | Louisville Dental Depot. |
| George W. Fels | 139 W. Sixth Street, Cincinnati, Ohio | Dental Depot. |

| NAME. | ADDRESS. | BUSINESS. |
|---|---|---|
| St. Louis Dental Mfg. Co | St. Louis, Mo | Dental Depot. |
| George C. Frye | Portland, Maine | Dental Depot. |
| J. B. Dunlevy | Pittsburgh, Pa | Dental Depot. |
| Minnesota Dental Mfg. Co | St. Paul, Minn. | |
| T. P. Wagoner | 64 Main Street, Knightstown, Ind | Dental Goods. |
| C. B. Woodward & Co | Fort Wayne, Ind. | Dental Depot. |
| J. E. Bodine & Co | Indianapolis, Ind | Dental Depot. |
| Illinois Dental Mfg. Co | 85 Fifth Ave., Chicago, Ill | Dental Supplies. |
| S. A. Crocker & Co | 119 W. Fifth St., Cincinnati, Ohio | Ohio Dental and Surg. Depot. |
| A. Grant | 917 Main St., Richmond, Va. | Dental Depot. |
| Robinson & Armstrong | Watertown, N. Y | Dental Supplies. |
| J. Durbin | Denver, Col | Dental Goods. |
| Charles Oehlmann | Quincy, Ill. | Dental Depot. |
| Morrison Bros | Nashville, Tenn | Dental Depot. |
| F. M. Allen, D.D.S | Birmingham, Ala | Dental Depot and Dentist. |
| W. C. Messinger | Hartford, Conn | Dental Supplies. |
| L. S. Kellner | Knoxville, Tenn | East Tennesee Dental Depot. |
| E. G. Fowler | Montgomery, Ala | Dental Depot. |
| Marshall Dental Mfg. Co | 309 Seventh Street, Des Moines, Iowa. | |
| Wm. M. Williams | Springfield, Mass | Dental Depot. |
| E. S. Holmes | Grand Rapids, Mich | Dentist. |
| E. J. Lewis | 1215 Pennsylvania Ave., Washington, D.C | Dealers in Dental Materials. |
| M. A. Spencer & Co. | 134 W. Seventh Street, Cincinnati, O. | Surg. and Dent. Instruments. |
| Buffalo Dental Mfg. Co | 587 and 589 Main Street, Buffalo, N. Y | Dental Depot. |
| R. I. Pearson & Co | 906 Grand Avenue, Kansas City, Mo | Surgical and Dental Depot. |
| Dr. C. E. Fitzgerald | Paris, Tenn | Dentist. |
| C. H. Hubbard | 44 Adelaide Street W., Toronto, Canada. | |
| S. B. Chandler Dent. Mfg. Co. | Toronto, Canada | Dental Goods. |

